# Comparative study on the dynamic properties of lightweight porous concrete

**DOI:** 10.1039/c8ra00082d

**Published:** 2018-04-18

**Authors:** Er-Lei Bai, Jin-Yu Xu, Song Lu, Ke-Xin Lin, Yi-Ming Zhang

**Affiliations:** Department of Airfield and Building Engineering, Air Force Engineering University Xi'an 710038 China baierlei647@163.com; College of Mechanics and Civil Architecture, Northwest Polytechnic University Xi'an 710072 China; Engineering Quality Supervision Station, The Sixth Air Defense Engineering Department Guangzhou 510052 China; Transport Barracks Section, Ninth Brigade of Air Force Radar Shijiazhuang 100720 China

## Abstract

In this paper, two types of lightweight porous concrete material, expanded polystyrene concrete (EPSC) and ceramics-cement based porous material (CCPM) have been prepared on the base of C60 concrete. The dynamic mechanical experiments of lightweight porous concretes have been carried out by *Φ* 100 mm split Hopkinson pressure bar (SHPB) improved by wave shaping technology. The dynamic properties, including strength properties, deformation properties, impact toughness and energy absorption properties, of lightweight porous concretes have been analyzed comparatively, and its application prospects have been discussed. The results show that the two types of lightweight porous concretes are strain rate sensitive. Dynamic compression strength increases with strain rate; the correlation between the peak strain, ultimate strain of lightweight porous concrete and strain rate can be expressed by quadratic polynomial; under the impact loading, the impact toughness of lightweight porous concretes increases with strain rate, the amount of absorbed energy increases with the average incident energy change rate, moreover, the relation between the impact toughness and strain rate, and that between the amount of absorbed energy and the average incident energy change rate can both be expressed by exponential functions; compared with EPSC, CCPM has better properties in terms of strength, deformation, impact toughness and energy absorption. Those advantages are more obvious with high strain rate. Therefore, CCPM has more vast application prospects in civil defense projects than EPSC.

## Abstract 2

In this paper, two types of lightweight porous concrete material, expanded polystyrene concrete (EPSC) and ceramics-cement based porous material (CCPM) have been prepared on the base of C60 concrete.

## Graphical abstract

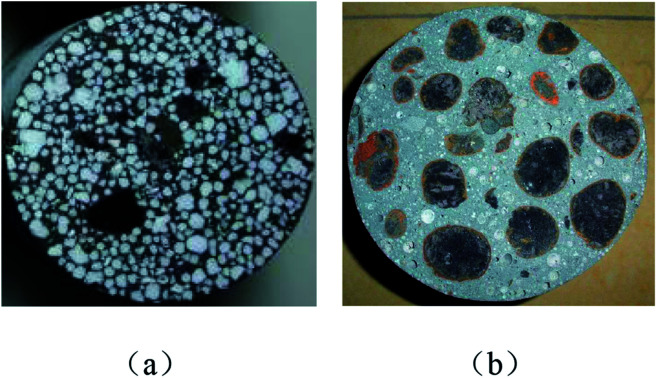

## 1 Introduction

Lightweight porous concrete is a kind of construction material. Owing to the addition of a large number of pores to the concrete, lightweight porous concrete, like autoclaved aerated concrete, ceramsite concrete (ceramsite is a kind of lightweight porous aggregate added into concrete), and expanded polystyrene concrete (EPSC), is light, heat preserving, heat isolating and porous. As a result, it has good prospects for application. Lightweight porous concrete belongs to the category of porous material. With a large number of pores, lightweight porous concrete has compressibility, and compressive stress platform, and its Poisson's ratio is almost zero. Those qualities make lightweight porous material an excellent energy-absorbing material.

Until now, a large number of research studies have been conducted with regard to the mechanical performance of porous material. For instance, Silva and Gibson conducted mechanical experiments on the porous materials and analyzed their deformation properties. According to their research, nonperiodic microstructure could lead to increasing more deformation and decreasing more strength compared with periodic microstructure.^1^ Wang conducted a certain research on the dynamic performance of porous material under impact loading. The results show that porous material could exhibit excellent performance in the resistance of explosive or impact loading.^2^ In view of the advantages in energy absorption, lightweight porous concretes have important application prospects in the civil defense project. EPSC^3–5^ is the most outstanding in performance according to present research situation. Ceramics-cement based porous material (CCPM) is a new kind of composite material, which possesses the double advantages of concrete material and porous material, and can play the superimposed effect of them. So far, the research on the dynamic performance of porous concrete is yet not much. Luo *et al.* studied the dynamic performance of porous concrete. The results indicated that this material possessed excellent dynamic performance, especially in the deformation performance.^6^ Therefore, as new materials in civil defense project, researches on the contrast of EPSC and CCPM have important application value in guidance of material selection of civil defense engineering. Besides, the related researches are also helpful to promote the development of porous materials.

In this paper, the expanded polystyrene (EPS) and ceramsite aggregate were used as the lightweight porous aggregate added into the matrix of C60 concrete. On the basis of this matrix, EPSC and CCPM have been prepared. The dynamic mechanical experiments of lightweight porous concretes have been carried out with *Φ* 100 mm split Hopkinson pressure bar (SHPB) improved by wave shaping technology; the mechanical parameters under different strain rates have been obtained; the dynamic properties of lightweight porous concretes, including strength property, deformation property, impact toughness and energy absorption properties have been analyzed contrastively, and its application prospects have been discussed.

## 2 Basic situation of the test

### 2.1 Matrix design

The raw materials used to prepare C60 ordinary concrete mainly include: cement, fly ash, sand, gravel, superplasticizer, silica fume, and water.

The basic property of each composition is listed as below: 42.5R ordinary portland cement; grade I fly ash (low calcium); medium sand: fineness modulus is 2.78, grading qualified, density is 2.63 g cm^−3^, bulk density is 1.50 kg L^−1^, silt content is 1.1%; crushed limestone: particle size is 5–20 mm, density is 2.70 g cm^−3^, bulk density is 1.62 kg L^−1^, silt content is 0.2%; superplasticizer: water reducing rate is 20%; silica fume: average particle size is 0.1–0.15 μm, specific surface area is 15–27 m^2^ g^−1^, SiO_2_ content is 85–95%.

The mixture ratio of C60 ordinary concrete is shown in the Table 1.

**Table 1:** The mixture ratio of C60 ordinary concrete

Cement (kg m^−3^)	Fly ash (kg m^−3^)	Silicon ash (kg m^−3^)	Superplasticizer (kg m^−3^)	Water (kg m^−3^)	Sand (kg m^−3^)	Gravel (kg m^−3^)
386	213.5	29.68	5.93	184	599	1069.5

### 2.2 Preparation of lightweight porous concrete

EPSC is a sort of composite material which adopt expanded polystyrene (EPS) particles to displace the sand and gravel aggregate under the condition of the same volume, and the volume ratio between sand and stone in the matrix is maintained. In this paper, EPSC with the EPS volume content of 50% has been prepared.

The mixture ratio of EPSC is shown in Table 2.

**Table 2:** The mixture ratio of EPSC

Cement (kg m^−3^)	Fly ash (kg m^−3^)	Silicon ash (kg m^−3^)	Superplasticizer (kg m^−3^)	Water (kg m^−3^)	Sand (kg m^−3^)	Gravel (kg m^−3^)	EPS (L)
386	213.5	29.68	5.93	184	122	219	500

The mixing system of EPSC: according to the characteristics of EPS, to improve its adhesion with cement paste and to prevent segregation and layering, after repeated trial mix, the mixing process should be as follows: firstly, mix fly ash, silicon ash, admixture, part of cement and water together to form a mortar with low water–cement ratio (30 s); then add EPS particles (30 s), and then add the rest of the water and cement, and mix for 30 s, and finally add sand, stone (120 s), stir into uniform mixture.

Ceramic aggregate includes alumina hollow ball (Al_2_O_3_ > 99%), compressive strength at normal temperature > 8MPa, four parts of particle size level (0.2–1.0 mm, 1.0–2.0 mm, 2.0–3.0 mm, 3.0–5.0 mm) are used and ceramsite (packing density is 510 kg m^−3^), cylinder pressure strength ≥ 1.5MPa, water absorption ≤ 15%, shape coefficient is “globular shape ≤ 1.6” (the ratio of the maximum size of aggregate particles to the minimum size of its middle section). CCPM has been prepared on the base of C60 ordinary concrete, which adopted alumina hollow ball and ceramsite to displace the sand and gravel aggregate completely, and the volume ratio between alumina hollow ball and ceramsite determined according to their particle size distribution. The mixture ratio of CCPM is presented in Table 3.

**Table 3:** The mixture ratio of CCPM

Cement (kg m^−3^)	Fly ash (kg m^−3^)	Silicon ash (kg m^−3^)	Superplasticizer (kg m^−3^)	Water (kg m^−3^)	Ceramsite (kg m^−3^)	Alumina hollow ball (kg m^−3^)
386	213.5	29.68	5.93	184	352	226

The mixing system of CCPM: mix superplasticizer and water up previously, and keep it for further use. Considering the characteristics of the alumina hollow balls and ceramsite, the mixing process should be as follows: the mixing process: (1) stir the micro-silica and half amount of the cement for 30 seconds. (2) Add three quarters of the prepared superplasticizer and continue to stir for 30 seconds. (3) Add the ceramsites and stir for another 30 seconds; (4) add the remaining superplasticizer and cement, stir the mixture for another 120 seconds, and the uniform mixture is made. Put the mixture out of the mixer, and manually blend it while sprinkling the alumina hollow balls.

### 2.3 Specimen and its basic characteristics

**Fig. 1:**
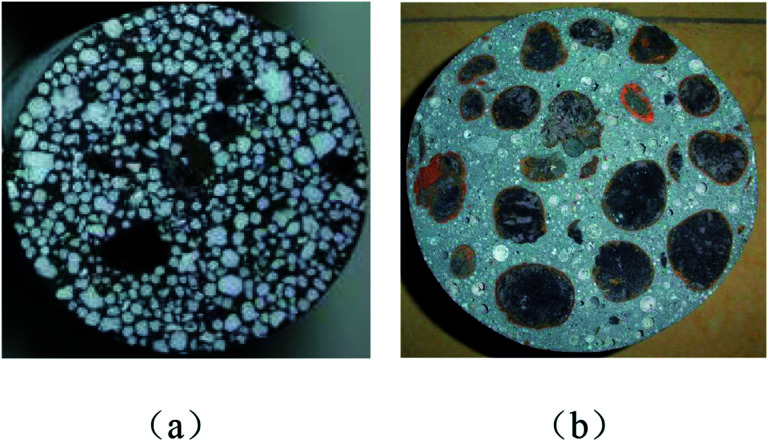
Cylindrical specimens. (a) EPSC, (b) CCPM.

According to the requirements of the test, fresh concrete is enclosed in the mould. To prevent water loss on the surface, it should be covered with plastic wrap. After 28 days of standard curing (*T* = 20 + 2 °C, relative humidity RH > 95%), take the specimens out and the cylindrical specimens used in the dynamic compression tests have been prepared. The cylindrical specimens should be polished to control their width and surface flatness, with geometry size of *Φ* 95 × 50 mm, as shown in Fig. 1.

The test shows that the quasi-static compressive strength of EPSC is 15.64 MPa, and the bulk density is 1494.71 kg m^−3^; the quasi-static compressive strength (*f*_c,s_) of CCPM is 18.27 MPa, and the bulk density is 1397 kg m^−3^.

## 3 Dynamic properties testing technology

### 3.1 SHPB test system

**Fig. 2:**
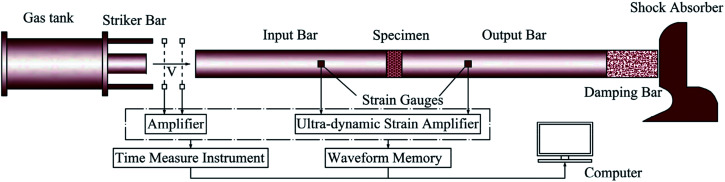
100 mm-diameter SHPB apparatus.

As is shown in Fig. 2, a 100 mm-diameter SHPB^7–9^ is used for testing, and this apparatus consists of main body, energy source and measurement systems.

The length of the projectile is 500 mm. The diameter of the compression bar is 100 mm. The input bar, transmission bar and absorbing bar are as long as 4.5 m, 2.5 m and 1.8 m respectively. They share the same standards as they are made 48 CrMoA, their elastic modulus is 210 GPa, Poisson's ratio is 0.25–0.3 and density is 7850 kg m^−3^.

The basic principle of SHPB testing is the propagation theory of elastic stress wave on the bar, and the test is based on the two following basic assumptions: (1) plane assumption: in the propagation process, each cross section of the elastic bar always keeps in a plane state. (2) Equal stress assumption: the stress in the specimen is equal everywhere. In the dynamic experiment, the specimens are placed between the input bar and output bar. When the striker bar is pulsed by the high pressure gas from gas tank, it will strike the input bar and form an elastic stress wave. Then, the elastic stress wave will spread through the input bar. When the wave reach at the interface between input bar and specimen, some wave will pass through the interface and go into the specimen, the rest will be reflected into the input bar. When the wave going into specimen reach at the interface between specimen and output bar, some wave will also be reflected into specimen and the rest will spread into the output bar. As a result, the wave in the specimen will be reflected back and forth and finally a state of equilibrium is reached.

### 3.2 Data processing method

Incident pulse *ε*_i_, reflected pulse *ε*_r_ and transmitted pulse *ε*_t_ can be recorded by the strain gauge on the bar. Based on the plane and equal stress assumption and the one-dimensional stress wave theory, the measurement data can be converted into strain rate (
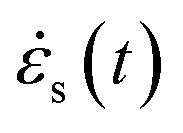
), stress (*σ*_s_(*t*)) and strain (*ε*_s_(*t*)), which can be expressed respectively as:1
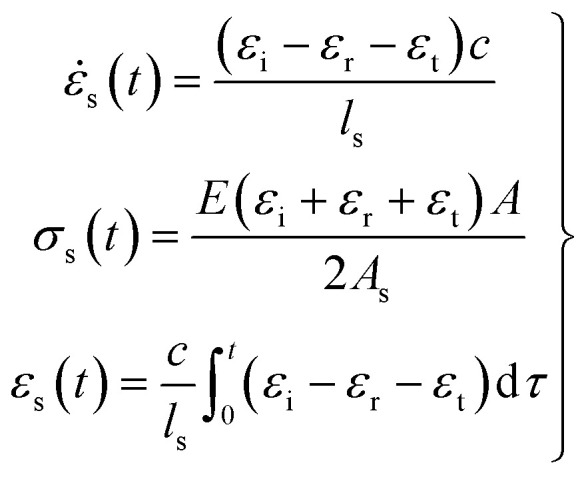
where *E* is the Young's modulus of bars, *c* is the wave velocity in bars; *A* and *A*_s_ are cross-sectional areas of bars and the specimen respectively; *l*_s_ is the original length of the specimen.

### 3.3 The key technology

**Fig. 3:**
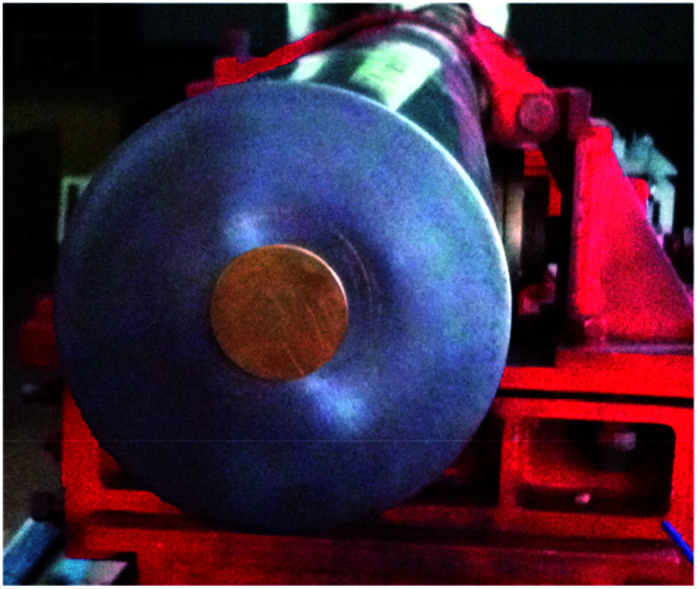
Pulse shaper used in SHPB apparatus.

In order to guarantee the validity of the test, H62 brass pulse shapers of different geometries *i.e.* 1 mm thickness and 20 mm, 22 mm, 25 mm, 27 mm and 30 mm diameter, as shown in Fig. 3, are designed to improve the SHPB test system.

Numerous studies have shown that wave shaping technology^10–13^ can achieve the following objectives: smooth waveform, eliminate the highly frequent oscillation of stress wave, reduce the wave dispersion in long distance transmission. As waveform grows wider, the rising edge of incident wave is extended, and the specimens have enough time to achieve uniform stress. As a result, constant strain rate loading can be realized.

## 4 Dynamic properties

### 4.1 Stress strain curve

**Fig. 4:**
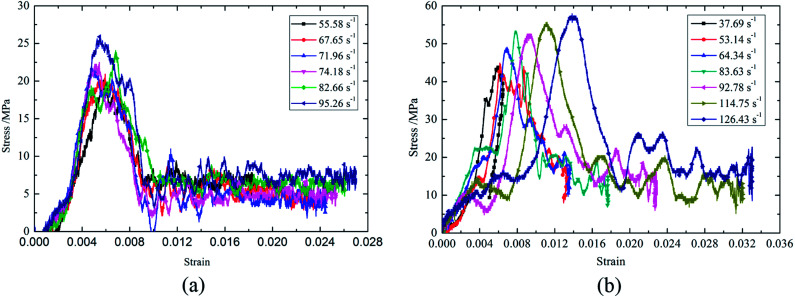
Stress–strain curves. (a) EPSC, (b) CCPM.

Fig. 4 shows dynamic compression stress–strain curve of lightweight porous concretes.

According to the Fig. 4, there are some similarities and differences between the stress strain curves of the two types of lightweight porous concretes: first of all, strain rate has an influence on the stress strain curve, and the stress strain curve changes with strain rate, but comparatively, strain rate has higher effect on the stress strain curve of CCPM than that of EPSC. That means in terms of stress strain curve, strain rate sensitivity of CCPM is stronger. Secondly, platform phenomenon appears in the stress strain curve, that's to say, as strain increases, the stress is not changed. The difference is that the platform phenomenon embodied in the stress strain curve of EPSC occurs only after the peak stress, but the platform phenomenon embodied in the stress strain curve of CCPM occurs both before and after peak stress.

### 4.2 Strength properties

**Fig. 5:**
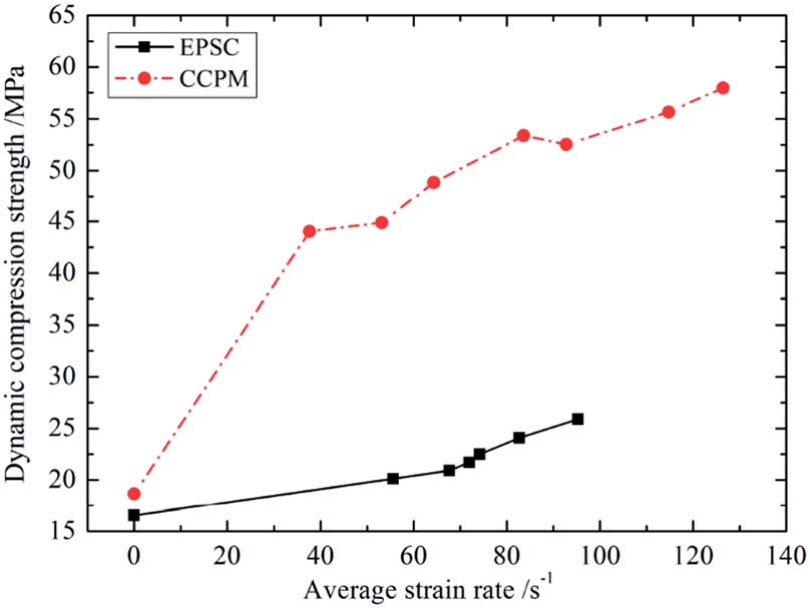
The relationship between *f*_c,d_ and 
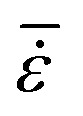
.

The dynamic compression strength *f*_c,d_ is defined as peak stress in the dynamic compression test. *f*_c,d_ is one of the major indicators reflecting dynamic strength of the material. Fig. 5 shows the *f*_c,d_ different strain rates.

As shown in Fig. 5, *f*_c,d_ of lightweight porous concrete increases with strain rate. The amplitude of *f*_c,d_ increases 1.2 times more than *f*_c,s_ does. This difference is more prominent when it comes to CCPM. When 
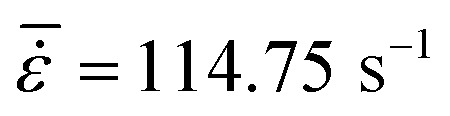
, the difference between *f*_c,d_ and *f*_c,s_ is 3.048 times and when 
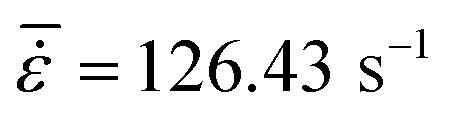
, it is 3.174 times. Thus it can be seen that, the two types of lightweight porous concrete are both strain rate sensitive. This point is consistent with the conclusions got by the scholars^14–17^ studying the dynamic mechanical performance of ordinary cement based composite material. It shows that cement based composite materials have some common characteristic, and it also testifies the accuracy of the test. The phenomenon that strength increases with strain rate can be called strain rate hardening effect, which is an integrated embodiment of Stefan effect^18^ and inertia effect.^19^

By comparison, the strength sensitivity of CCPM is stronger than that of EPSC. This difference is particularly prominent in low strain rate. When 
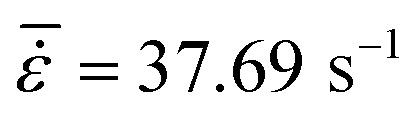
, *f*_c,d_ of CCPM is 2.41 times *f*_c,s_, while when 
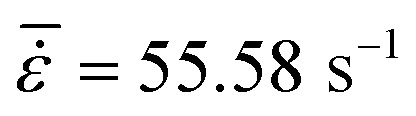
, *f*_c,d_ of EPSC is only 1.288 times *f*_c,s_. Thus it can be seen, when applied to projects withstanding impact and explosion loading, CCPM has obvious advantages in strength. The more the impact intensity is, the higher the strength.

### 4.3 Deformation properties

**Fig. 6:**
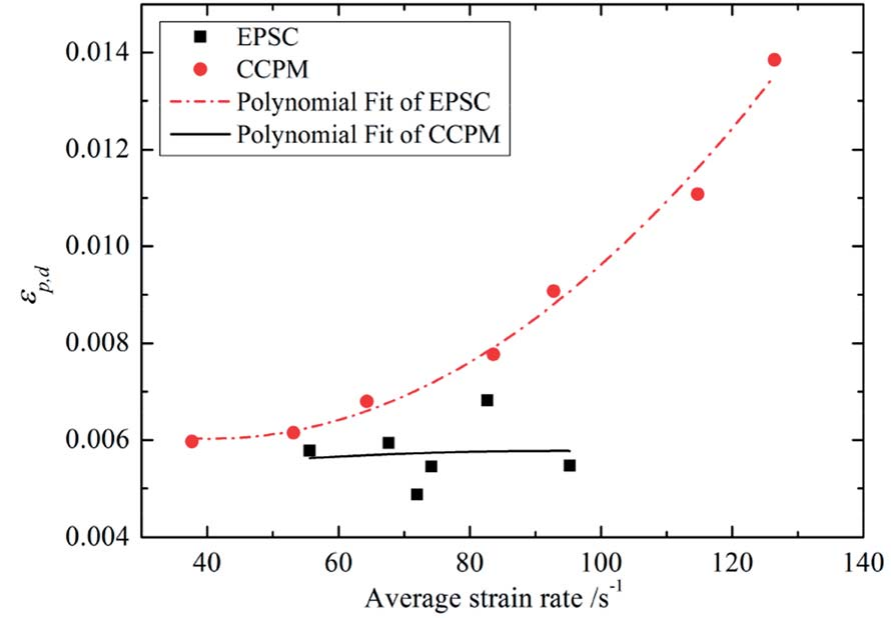
The relationship between *ε*_p,d_ and 
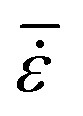
.

Peak strain and ultimate strain are important indexes of deformation characteristics. Peak strain in the dynamic compression test is identified as *ε*_p,d_ and ultimate strain is identified as *ε*_L,d_. Fig. 6 shows the relationship between *ε*_p,d_ and average strain rate.

The figure shows that *ε*_p,d_ of lightweight porous concretes changes with strain rate. The *ε*_p,d_ of CCPM increases with strain rate, while the *ε*_p,d_ of EPSC decreases with the increase of strain rate. Through analysis, the correlation between *ε*_p,d_ and 
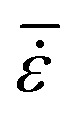
 can be expressed by a quadratic polynomial, as shown in formula (2):2
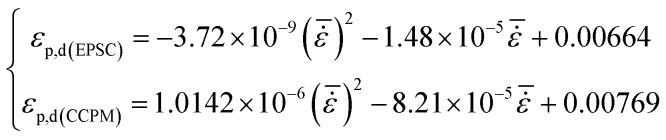


The formula above is similar to the experience formulas obtained by Tedesco^20^ and Dilger.^21^

By comparison, it shows that when strain rate is low, the peak strains of EPSC and CCPM appear to be a little different. But as strain rate increases, the difference between the peak strains of the two types of lightweight porous concrete grows bigger and bigger. When 
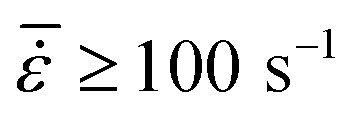
, *ε*_p,d_ of CCPM is more than 1.5 times that of EPSC. The reasons can be listed as follows. When the strain rate is low, the deformation degrees of the porous aggregates added in EPSC and CCPM are similar as the stress reach at the peak. As the strain rate increases, the deformation of the porous aggregate in CCPM will get larger, while the deformation of the porous aggregate in EPSC will weaken. That is because the porous aggregate in EPSC is inlaid in the stone system, the stress in the stone system will get larger with the increase of strain rate. Owing to the resistance of stone system, the stress in the porous aggregate will decrease, and the deformation will also be lowered accordingly.

**Fig. 7:**
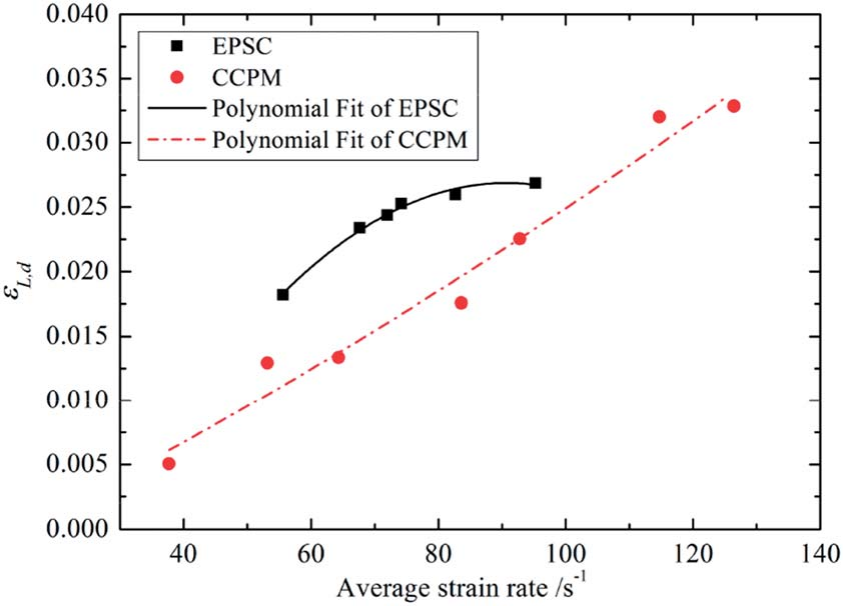
The relationship between *ε*_L,d_ and 
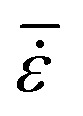
.

Fig. 7 shows the relationship between *ε*_L,d_ and average strain rate.

The Fig. 7 shows that *ε*_L,d_ of lightweight porous concretes increases with strain rate, and the correlation between *ε*_L,d_ and 
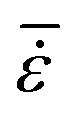
 can be expressed by a quadratic polynomial, as shown in formula (3):3
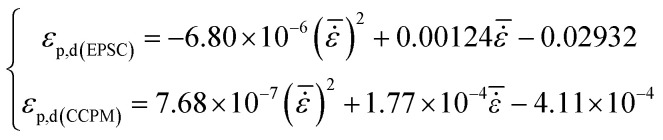


By comparison, it shows that under low strain rate, the *ε*_L,d_ of EPSC is higher than that of CCPM. But, from the analysis of correlation curve, the *ε*_L,d_ of CCPM and EPSC increase with strain rate. As strain rate increases, the *ε*_L,d_ of CCPM increases faster and faster while that of EPSC increases slower and slower. So it can be concluded that under high strain rate, the *ε*_L,d_ of CCPM is higher than that of EPSC. And the higher the strain rate is, the more obvious this trend becomes. At present, there are few studies reporting the ultimate strain performance of porous concrete. Therefore, the results concerning about the ultimate strain performance is worthy addition to the dynamic characteristics of porous concrete.

Overall, the deformation characteristic of CCPM is better than that of EPSC. Moreover, this phenomenon becomes more obvious as the strain rate increases.

### 4.4 Impact toughness

**Fig. 8:**
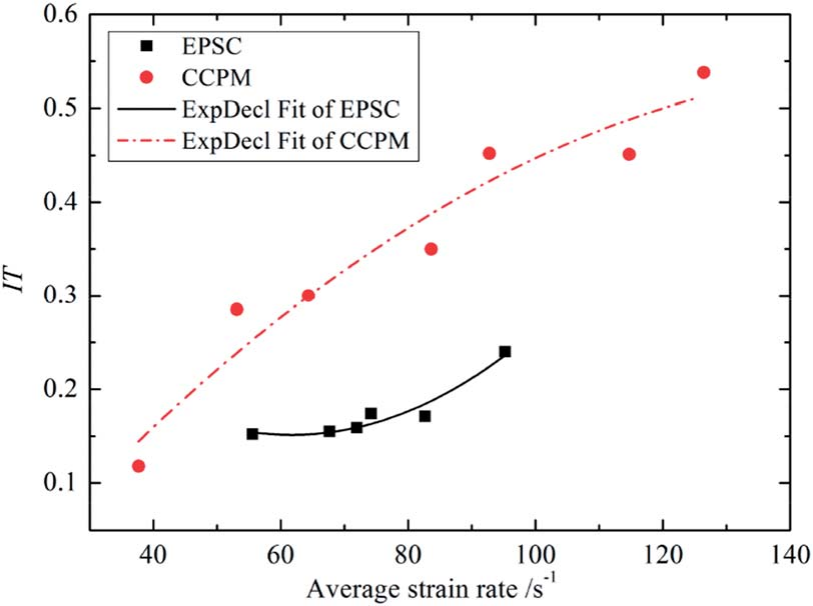
The relationship between IT and 
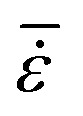
.

Impact toughness is not only related to the strength of the material, but also depends on the deformation at the moment when the material breaks. It can be expressed by the area enclosed by the stress–strain curve and axial strain.^23^ The impact toughness of lightweight porous concrete can be identified as IT. Fig. 8 shows the relationship between IT and average strain rate.

The Fig. 8 shows that the impact toughness of lightweight porous concretes increase with strain rate, and the correlation between the IT and 
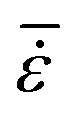
 can be expressed by an exponential function, as shown in formula (4):4
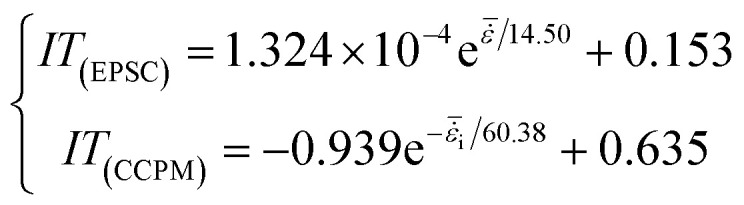


By comparison, the IT of CCPM under different strain rates is much higher than that of EPSC, and as strain rate increases, the gap between IT of CCPM and EPSC increases first and then decreases. When the average strain rate is 82 s^−1^, the gap reaches a maximum.

Comprehensive analysis shows that the impact toughness of CCPM is better than that of EPSC. It can also be concluded through the analysis of strength and deformation properties.

### 4.5 Energy absorption properties

*W*_i_(*t*), *W*_r_(*t*), *W*_t_(*t*) are respectively defined as incident wave energy, reflected wave energy and transmitted wave energy. The expressions are shown as below:5
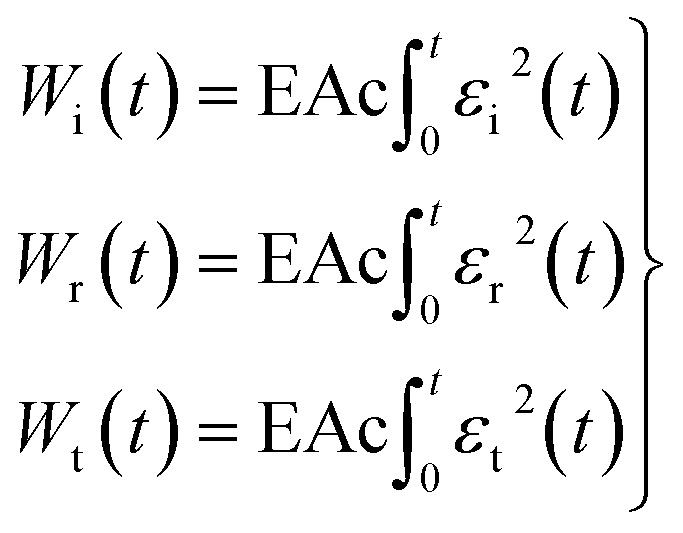


The average incident energy change rate *W̄*_i_^22^ is defined as in formula (6):6
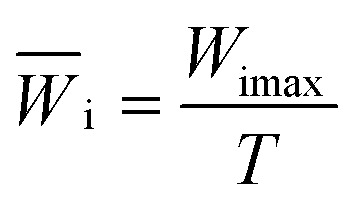
where *T* is the total duration time of incident wave.

Assuming energy loss on interface between specimen and incident bar or transmitted bar is neglected, according to the conservation of energy; the energy absorbed by the broken specimen can be expressed as follows:

7 *W*_s_(*t*) = *W*_i_(*t*) − *W*_r_(*t*) − *W*_t_(*t*)

The energy absorption at the moment that specimen gets completely destroyed can be defined as the total energy absorption of specimen *W*_smax_.

**Fig. 9:**
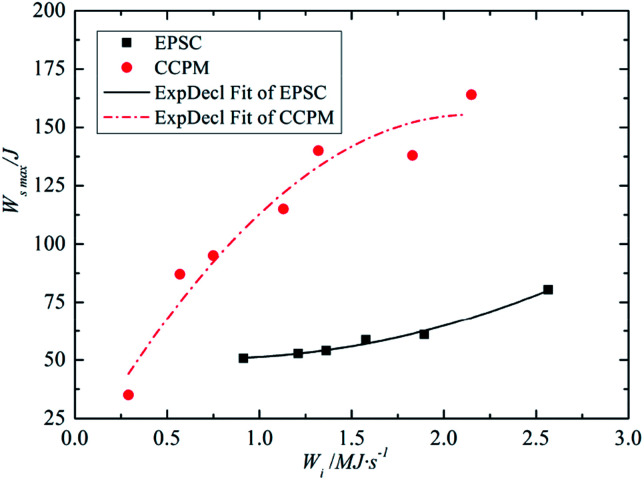
The relationship between *W*_smax_ and *W̄*_i_.

Fig. 9 shows the relationship between the energy absorption *W*_smax_ and the average incident energy change rate.

According to the analysis of the Fig. 9, the relation between the *W*_smax_ of lightweight porous concrete and *W̄*_i_ is consistent with the change law between IT and *W̄*_i_. That's to say, *W*_smax_ increases with *W̄*_i_, and the relationship between *W*_smax_ and *W̄*_i_ can be expressed by an exponential function, as shown in formula (8).8
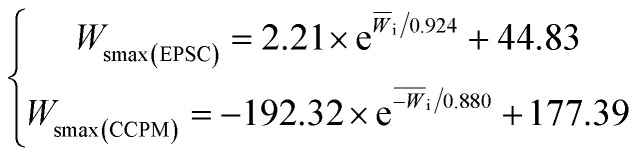


By comparison, the energy absorption of CCPM are much higher than that of EPSC under the conditions of every incident energy change rate, that's especially prominent in high strain rate. That is because the deformation characteristic of CCPM is better than that of EPSC, especially in high strain rate. Thus it can be concluded that the energy absorption characteristic of CCPM is better than that of EPSC.

### 4.6 Application and mechanism analysis

Researches^3,24,25^ at home and abroad show that EPSC has excellent mechanical properties. But from the analysis above, in terms of strength, deformation, impact toughness and energy characteristics, CCPM is more excellent than EPSC, and this advantage is more obvious in high strain rate. In addition, the density of CCPM is lower than that of EPSC. Therefore, CCPM has a broader application prospect in civil defense engineering than EPSC. It's necessary to continue to develop the study on CCPM, which has important theoretical and practical value.

The excellent properties of lightweight porous concrete are macro performance of mechanical properties, which is closely connected with microstructure. Owing to the addition of EPS or porous ceramsite and alumina hollow ball, the internal part of lightweight porous concrete is full of holes, and all the internal holes are combined into the hole structure. All of the mechanical properties of lightweight porous concrete, therefore, are the combined characteristics of substrate material and hole structure. When subjected to impact loading, hole structure collapses and closes, then lightweight porous concrete can have a long trip under constant stress, and absorb a lot of energy, which can also be derived from the analysis of the stress strain curve.

Although EPSC and CCPM are lightweight porous concrete, the mechanical properties of CCPM are better than those of EPSC, which has to give the credit to the uniqueness of CCPM's inner hole structure. The holes of CCPM include tiny holes within ceramsite and the larger hollows within alumina hollow balls, and these holes have the distribution in different particle size range. However the holes of EPSC mainly include EPS particles, so the particle size is relatively single, moreover, holes of CCPM are more than those of EPSC; Compared to EPSC, the hole structures of CCPM collapse hierarchically under the loading, and the compressed space is larger, so the mechanical properties of CCPM are better than those of EPSC.

## 5 Conclusions

The dynamic mechanical experiments of the two types of lightweight porous concrete (EPSC and CCPM) have been carried out with *Φ* 100 mm SHPB device improved by wave shaping technology. The dynamic properties of lightweight porous concretes, including strength property, deformation property, energy absorption properties and impact toughness, have been analyzed contrastively, and its application prospects have also been discussed.

The two types of lightweight porous concrete are strain rate sensitive materials. The mechanical properties, including strength, peak strain, ultimate strain and impact toughness, are change with the increase of strain rate. Under impact loading, the total energy absorption of lightweight porous concrete increases with incident energy change rate, and the relationship between them is expressed by an exponential function. Compared with EPSC, CCPM is more excellent in terms of strength, deformation, impact toughness and energy, and the advantage is more outstanding under high strain rate.

In view of the fact that the mechanical properties of CCPM are better than those of EPSC, CCPM has a broader application prospect in civil defense engineering than EPSC.

## Conflicts of interest

There are no conflicts to declare.

## Supplementary Material
